# Improving the default data analysis workflow for large autoimmune biomarker discovery studies with ProtoArrays

**DOI:** 10.1002/pmic.201200518

**Published:** 2013-06-20

**Authors:** Michael Turewicz, Caroline May, Maike Ahrens, Dirk Woitalla, Ralf Gold, Swaantje Casjens, Beate Pesch, Thomas Brüning, Helmut E Meyer, Eckhard Nordhoff, Miriam Böckmann, Christian Stephan, Martin Eisenacher

**Affiliations:** 1 Medizinisches Proteom-Center, Ruhr-University BochumBochum, Germany; 2 Neurological Clinic, St. Josef-Hospital, Ruhr-University BochumBochum, Germany; 3 Institute for Prevention and Occupational Medicine of the German Social Accident Insurance, Institute of the Ruhr-University Bochum (IPA)Bochum, Germany

**Keywords:** Autoantibodies, Bioinformatics, Biological markers, Parkinson’s disease, Protein array analysis

## Abstract

Contemporary protein microarrays such as the ProtoArray® are used for autoimmune antibody screening studies to discover biomarker panels. For ProtoArray data analysis, the software Prospector and a default workflow are suggested by the manufacturer. While analyzing a large data set of a discovery study for diagnostic biomarkers of the Parkinson’s disease (ParkCHIP), we have revealed the need for distinct improvements of the suggested workflow concerning raw data acquisition, normalization and preselection method availability, batch effects, feature selection, and feature validation. In this work, appropriate improvements of the default workflow are proposed. It is shown that completely automatic data acquisition as a batch, a re-implementation of Prospector’s pre-selection method, multivariate or hybrid feature selection, and validation of the selected protein panel using an independent test set define in combination an improved workflow for large studies.

Contemporary protein microarrays are used for autoimmune profiling studies that aim to discover biomarker panels for potential autoimmune disorders by discriminating between persons who are categorized by disease status, severity of disease, or other factors. The ProtoArray® v5.0 provided by Life Technologies (Carlsbad, CA, USA) with about 9500 protein features spotted on each array is the leading platform in this area of research.

The vendor provides some recommendations (default workflow) and the free software Prospector (current version 5.2.1) for the analysis of ProtoArray autoimmune profiling data in gpr (GenePix results) file format. On the one hand, Prospector features an advantageous (subgroup-sensitive) univariate feature selection method for two-group discrimination (minimum M Statistic, “M Score” 1) as well as a ProtoArray-specific normalization approach (robust linear model 2). On the other hand, Prospector and the default workflow show some shortcomings that are fatal especially for studies that are large with regard to the technical workflow (e.g. group sizes >30 each). In this work, these shortcomings are discussed and solutions to improve the default workflow are proposed with reference to an exemplary large data set.

In the exemplary Parkinson’s disease (PD) study (“ParkCHIP,” a ProtoArray study that we have conducted at the Medizinisches Proteom-Center, to be published), 216 ProtoArrays have been incubated with sera from three clinical groups (72 PD cases, 72 healthy controls (HC), and 72 disease controls (DC), i.e. cases of other neurodegenerative and autoimmune diseases) to find evidence that PD is associated with a specific panel of autoimmune antibodies that can be used as diagnostic biomarkers (hypothesis corroborated by literature, especially 3). All samples have been collected at the Neurological Clinic of the St. Josef Hospital in Bochum and were 1:1:1 frequency-matched by age and gender. ProtoArrays are produced in lots (production lots) consisting of up to about 160 arrays each. Thus, this study was too large for a single lot and it had to be distributed among two lots (“lot1” and “lot2”).

**First improvement** – The recommended raw data acquisition with the semiautomatic workflow provided by the Software GenePix Pro 6 (Molecular Devices, Sunnyvale, CA, USA) is very time consuming and not reliable. Due to the manual steps of grid positioning (stored in gal files, i.e. GenePix Array Lists) and grid alignment correction, additional variance comprises the variation between and within subjects. Because one single person needs up to 30 min per slide, the processing of arrays is limited to 20 arrays per day (approximately 11 days/216 arrays), which makes the semiautomatic approach not feasible for large studies. Thus, reliable and fully automatic batch workflows should be used. Unfortunately, the automatic raw data acquisition workflow provided by GenePix Pro mostly fails to find all spots correctly. As a solution, the reliable batch mode of the alternative software StrixAluco 3.0 (Strix Diagnostics, Berlin, Germany) can be used to acquire all raw data in 1 day automatically without additional variance.

**Second improvement** – There is only a 32-bit version of Prospector available that does not run on 64-bit machines and cannot process a two-group comparison with more than 30 arrays per group (“out-of-memory” errors). This is fatal because Prospector is the only software providing the advantageous M Score. After manufacturer contact, we had a preliminary beta version of the 64-bit implementation for the ParkCHIP study. Alternatively, M Score can be reimplemented in R (4 http://www.r-project.org/) and raw data preprocessing can be performed using a convenient R package (e.g. limma 5, http://www.bioconductor.org/).

**Third improvement** – There is no solution for batch effects (i.e. systematic error caused by microarray processing in batches 6, 7) concerning production lots (here, “batch effects”) that can arise due to concentration differences in protein spots and other different spotting conditions. Batch effects are a severe methodological shortcoming in large biomarker studies using more than one lot, also when incorporating data from different labs or when pooling data from other studies.

Some ProtoArray studies ignore the lot problem and may thus report false-positive findings 8, 9. We were able to reanalyze those original data (Gene Expression Omnibus records “GSE29654” and “GSE29676,” http://www.ncbi.nlm.nih.gov/geo/) regarding this assumption. For example, in 8 there is a serious bias concerning the unequal distribution of clinical classes between the lots, because all their PD cases were processed with one lot and all controls with another. Therefore, the ten biomarker candidates proposed in 8 may be primary differential concerning their lots. In 9, there is a similar bias.

To solve the batch-effect problem, two approaches have been adopted for the ParkCHIP study. First, the study has been set up with the guideline to distribute the three groups equally among the production lots. Moreover, the arrays have been distributed equally among all processing steps and days in the lab to minimize sources of bias such as weather, time, technical and physicochemical factors, and the variation between or within subjects. Second, all lots discriminating protein features have been discarded (see the paragraph below). Alternatively, a computational adjustment for batch effects can be performed 6, 7.

**Fourth improvement** – Prospector provides only a univariate biomarker candidate selection approach (M Score) and has no multivariate selection capabilities 10. This is problematic because M Scores do not represent *p*-values, they are not adjusted for multiple testing, and there is no hint where to set an objective M Score threshold. Furthermore, this method ignores multivariate feature relations (two features having poorer scores may be superior in combination to the best two features 11). For the ParkCHIP study, the advantages of automatic univariate (fast) and multivariate wrapper (multivariate feature evaluation, interaction with classifier) methods have been combined with manual selection in a “hybrid feature selection” approach. Moreover, this approach solves the batch-effect and the multiclass problem (simultaneous discrimination of three or more clinical groups).

After raw data quality management and preprocessing (quantile and loess normalizations, respectively, performed with the R package limma), several two-group comparisons have been performed using Prospector. These comparisons were as follows: HC versus PD, DC versus PD, HC versus DC, and lot1 versus lot2 (each comparison with quantile and loess normalized data). For biomarker candidate selection, an ensemble selection scheme composed of “score voting,” “manual voting,” “manual selection,” and “automatic selection” has been conducted. Score votes were, for example, “1” for M Score <0.05 concerning HC versus PD and DC versus PD as well as for M Score ≥0.05 concerning HC versus DC and M score ≥0.00001 concerning lot1 versus lot2 or “0” otherwise. Manual voting was based on fluorescence intensity plots (one for each protein). Five persons have inspected these plots and rated the corresponding proteins as differential (by voting with “1” or “0”). Proteins fulfilling vote sum thresholds for certain subsets of manual or score votes, respectively, have been preselected. These “preselection rules” as a whole basically ensured the inclusion of proteins discriminating HC and PD as well as DC and PD and the exclusion of proteins discriminating HC and DC or lot1 and lot2 (tackling multiclass and lot problem). The resulting list containing 215 preselected proteins has been further narrowed down to 22 proteins by manual selection, that is, by setting an overall voting sum threshold (score voting sum + manual voting sum). Additionally, an automatic selection has been applied to the 215 preselected proteins. A multivariate feature selection wrapper approach 10, 12 has been implemented providing an evolutionary algorithm 13 as wrapping procedure and the random forest (“RF,” R package: randomForest) 14, 15 as wrapped classifier. After six runs with 100 000 iterations each, 14 additional proteins (distinct to the 22 obtained from final manual selection, actually there were 18) have been returned. The results of manual and automatic selection have been combined to a final biomarker panel containing 36 candidate proteins that discriminate the three groups DC, HC, and PD. The basic idea of the whole biomarker candidate selection procedure is outlined in Fig. 1 and the superiority of this method compared to the default workflow is shown in the Fifth improvement section.

**Figure 1:**
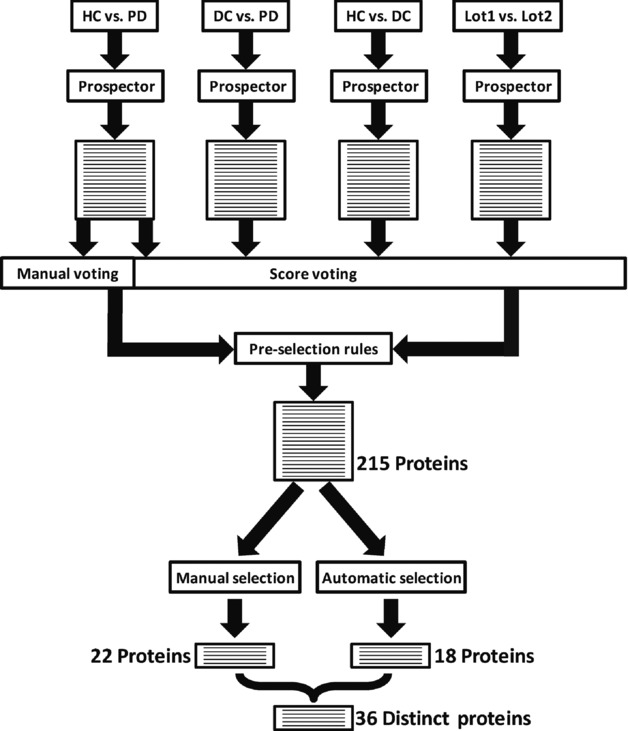
The basic idea of the hybrid procedure for the selection of candidate proteins, which has been performed in the ParkCHIP study, is shown. As first step, several two-group comparisons (HC vs. PD, DC vs. PD, HC vs. DC sera and lot1 vs. lot2) have been performed using the software Prospector. After rating all proteins by manual and score voting, preselection rules have been applied to them, and resulted in 215 preliminary biomarker candidates. This set has been further narrowed down by manual and automatic selection. Finally, the resulting lists containing 22 and 18 proteins, respectively, have been assembled to the final biomarker candidate list containing 36 distinct candidate proteins.

**Fifth improvement** – With the proposed default workflow, there is no reliable and bias-free strategy to validate the selected biomarkers with independent data (i.e. independent test set classification). To validate the 36 ParkCHIP candidates computationally, all samples of the reduced data set (containing 36 candidates only) PD versus “HC + DC” (combined group consisting of HC and DC) have been split randomly into a classifier training (2/3) and test set (1/3) conserving the respective experimental group proportions. Then, an RF has been trained using the training set. Subsequently, this RF has been applied to the test set to estimate the classification accuracy. The whole procedure including random splitting has been repeated ten times. Finally, the average of the ten subrun accuracies has been computed to assess the overall performance of the 36 biomarker candidates. To compare the results of the hybrid selection with the default workflow, additionally, ten new test and training set pairs have been resampled, for each training set 36 proteins with the best M Scores have been selected (“M Score only” selection) and an RF has been trained using the respective 36 features to classify the corresponding test set (see Fig. 2). The average accuracy has been computed also for these ten subruns.

**Figure 2:**
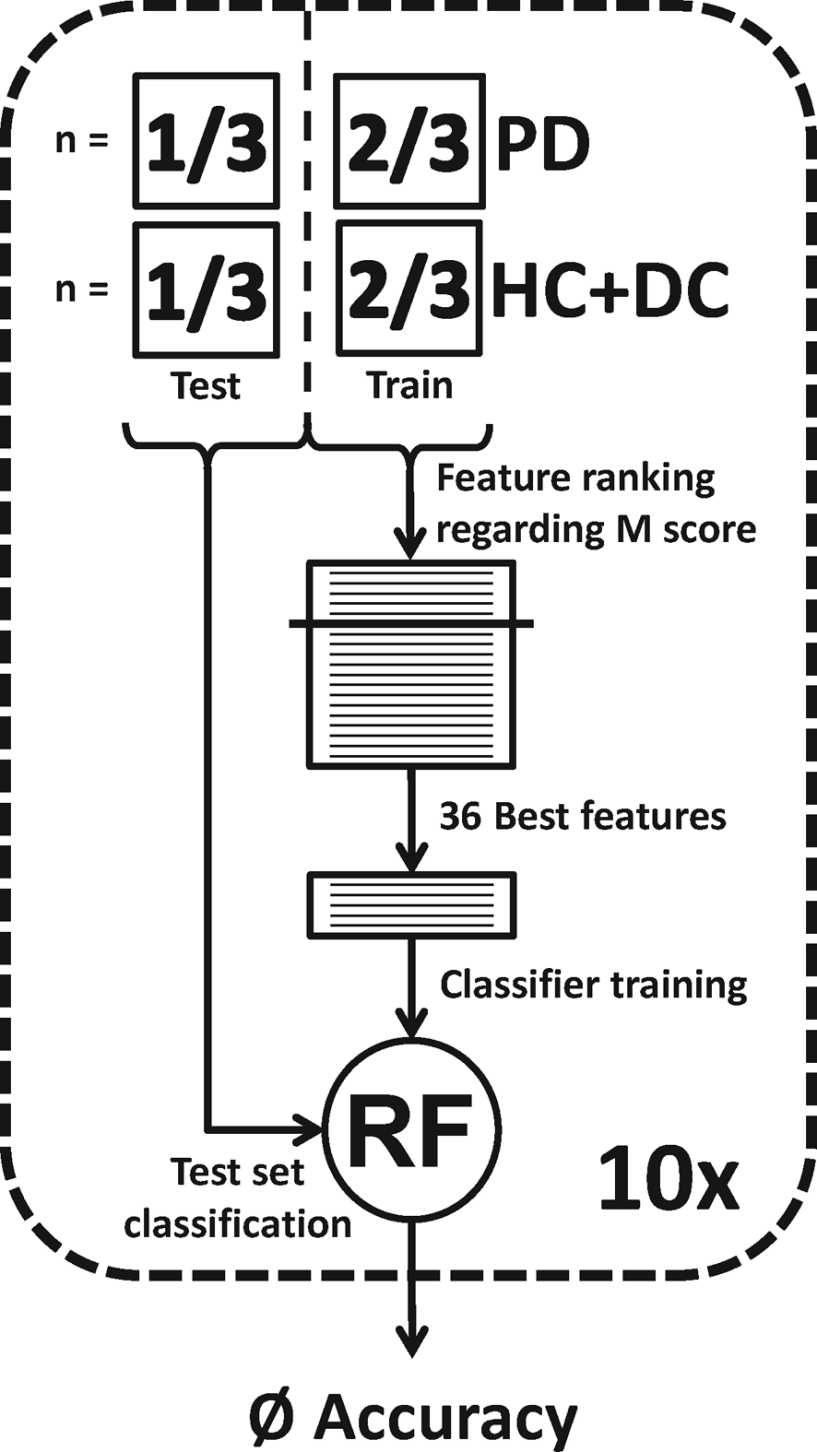
To validate the “M Score only” selection, ten new test and training set pairs have been resampled for the discrimination of PD cases and nonaffected subjects (PD and “HC + DC”). For each training set, the best 36 proteins (concerning M Score) have been selected and an RF classifier has been trained using the respective 36 features to classify the corresponding test set. Finally, the average accuracy for these ten subruns has been computed.

As a result (see Table 1) of the hybrid selection validation, the average classification accuracy values were 74.5% for the training set and 73.5% for the test set. Moreover, the variation in accuracy values of subruns was small, ranging from 74.0 to 74.8% for the training set and from 69.5 to 77.2% for the test set. In contrast, the validation results of the “M Score only” selection were very divergent. On the one hand, perfect training set classification in all subruns has been performed (all accuracies: 100%). On the other hand, the test set accuracies (ranging from 51.4 to 65.3%, average: 58.25%) amount to a classification slightly better than chance (see Table 1). Thus, there is obvious evidence for overfitting that has been caused by a fallacious univariate feature selection of the default workflow (perfect feature panel for the training set—poor panel for new data). The hybrid feature selection approach finds a more general feature panel. Hence, it is an improvement of the default workflow. Additionally, corresponding results for a large Alzheimer’s disease data set (50 Alzheimer’s disease vs. 40 HC vs. 59 DC, “GSE29676” 9) are outlined in Table 1. This study has been conducted using three lots without distributing the groups equally among the production lots. Hence, there are probably more serious batch effects than in the ParkCHIP data.

**Table:** For Parkinson’s (ParkCHIP) and Alzheimer’s disease data (GEO record GSE29676), particular test and training set classification accuracies for ten subruns of “M score only” selection and “hybrid” selection as well as on average are shown

	Parkinson’s disease				Alzheimer’s disease			
	M Score only		Hybrid		M Score only		Hybrid	
Subrun	Train	Test	Train	Test	Train	Test	Train	Test
1	100	60.1	74.5	74.9	100	75.6	83.9	84.8
2	100	55.3	74.6	77.2	100	79.6	82.6	83.9
3	100	54.9	74.5	71.2	100	81.6	83.9	78.9
4	100	51.4	74	72.4	100	77.6	83.9	79.9
5	100	55.7	74.5	73.5	100	75.5	83.9	75.7
6	100	56.7	74.8	73.2	100	87.6	83.2	77.3
7	100	58.2	74.5	73.8	100	80.8	83.2	91.1
8	100	62.1	74.4	69.5	100	79.2	83.9	83.8
9	100	65.3	74.7	74.5	100	74.4	83.9	83.4
10	100	62.8	74.7	74.5	100	81.0	83.9	87.2
Average	100	58.25	74.5	73.5	100	79.3	83.6	82.6

These exemplary results show that feature validation is indispensable. The default workflow with Prospector provides no feature validation. Thus, incorporating training and test set splits is a crucial improvement to obtain reliable biomarker panels. Alternatively, other validation methods such as k-fold cross-validation or bootstrapping can be used.

To sum up, it has been shown that the default workflow proposed by the ProtoArray manufacturer needs to be improved. The suggested raw data acquisition is time consuming and not reliable, the officially released version of Prospector cannot process large studies, there is no solution for the batch-effect problem, Prospector’s univariate feature selection strategy fails to select a generally suitable feature subset, and there are no capabilities to validate the selected biomarker candidate panel. In this work, straightforward solutions for these issues have been proposed. For raw data acquisition, an automatic and reliable batch mode should be used; to use the advantageous M Score, the 64-bit Prospector should be requested or M Score should be reimplemented. A multivariate or hybrid feature selection should be applied and the selected feature panel should be validated using an independent test set. In combination, these solutions define an improved workflow for the biomarker discovery with ProtoArrays.

## Abbreviations

DC: disease controls
HC: healthy controls
M Score: minimum M Statistic
PD: Parkinson’s disease
RF: random forest
